# Glycans as Key Checkpoints of T Cell Activity and Function

**DOI:** 10.3389/fimmu.2018.02754

**Published:** 2018-11-27

**Authors:** Márcia S. Pereira, Inês Alves, Manuel Vicente, Ana Campar, Mariana C. Silva, Nuno A. Padrão, Vanda Pinto, Ângela Fernandes, Ana M. Dias, Salomé S. Pinho

**Affiliations:** ^1^Institute of Molecular Pathology and Immunology of the University of Porto (IPATIMUP) Porto, Portugal; ^2^Institute for Research and Innovation in Health (I3S) Porto, Portugal; ^3^Institute of Biomedical Sciences of Abel Salazar, University of Porto Porto, Portugal; ^4^Medical Faculty, University of Porto Porto, Portugal; ^5^Centro Hospitalar do Porto Porto, Portugal

**Keywords:** *N*-glycosylation, glycans, T cells, immune response, autoimmunity, self-tolerance

## Abstract

The immune system is highly controlled and fine-tuned by glycosylation, through the addition of a diversity of carbohydrates structures (glycans) to virtually all immune cell receptors. Despite a relative backlog in understanding the importance of glycans in the immune system, due to its inherent complexity, remarkable findings have been highlighting the essential contributions of glycosylation in the regulation of both innate and adaptive immune responses with important implications in the pathogenesis of major diseases such as autoimmunity and cancer. Glycans are implicated in fundamental cellular and molecular processes that regulate both stimulatory and inhibitory immune pathways. Besides being actively involved in pathogen recognition through interaction with glycan-binding proteins (such as C-type lectins), glycans have been also shown to regulate key pathophysiological steps within T cell biology such as T cell development and thymocyte selection; T cell activity and signaling as well as T cell differentiation and proliferation. These effects of glycans in T cells functions highlight their importance as determinants of either self-tolerance or T cell hyper-responsiveness which ultimately might be implicated in the creation of tolerogenic pathways in cancer or loss of immunological tolerance in autoimmunity. This review discusses how specific glycans (with a focus on *N*-linked glycans) act as regulators of T cell biology and their implications in disease.

## Introduction

**Figure 1:**
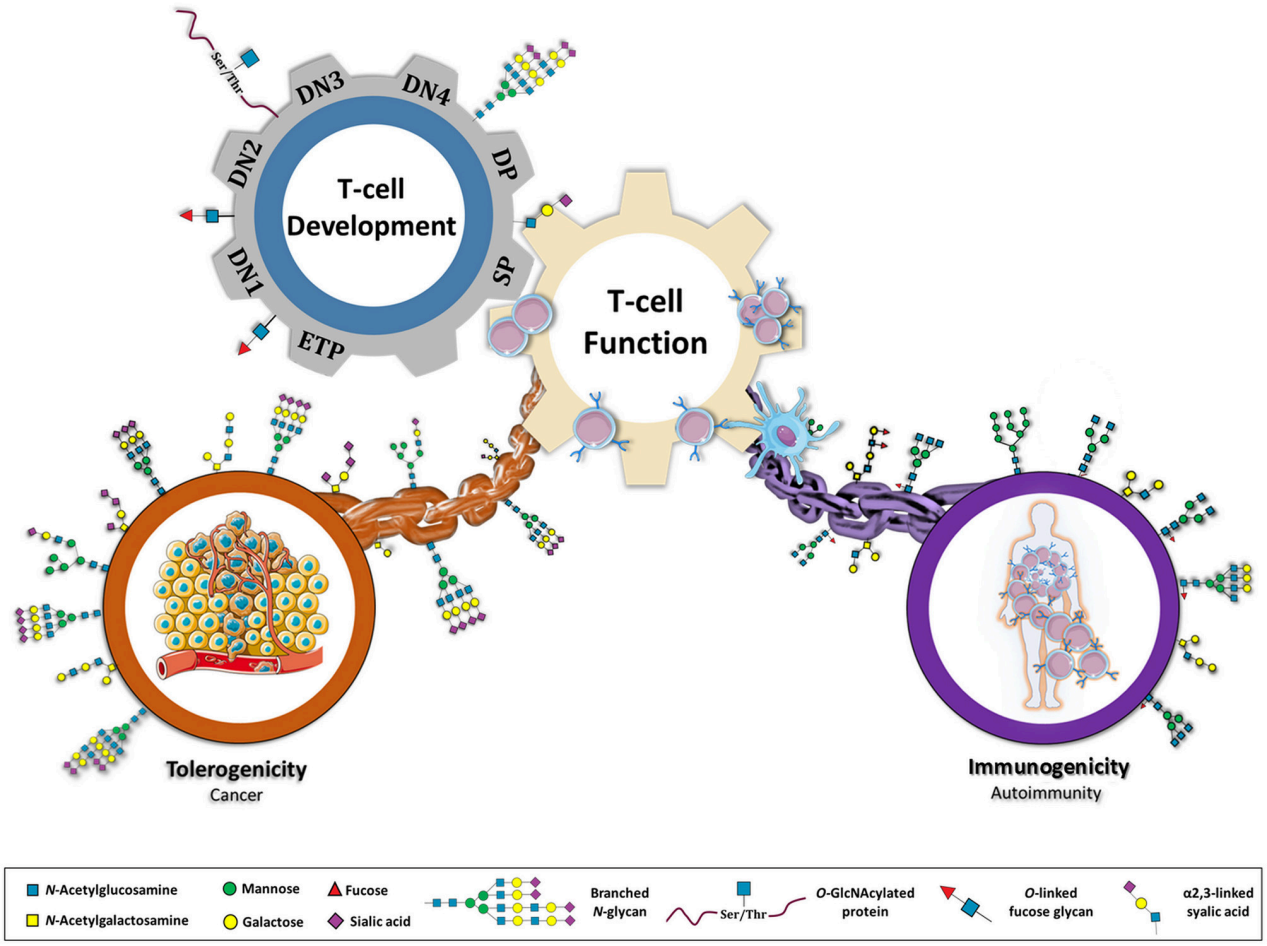
Glycans as a major connective chain that controls T cell response in either a tolerogenic or immunostimulatory scenario. Glycosylation appears to be central in regulating several steps of a T cell's life. During T cell development, different population of T cells (ETP, early thymocyte progenitor; DN1, 2, 3, and 4, double negative; DP, double positive; SP, single positive) display specific glycosylation patterns. The normal glycosylation of SP population results in an educated T cell function. However, by genetic, environmental or metabolic constrains, T cell glycosylation can be compromised re-directing immune system toward an immunostimulatory or tolerogenic response. Glycans are proposed here as key players in immune-unbalanced diseases, such as autoimmunity and cancer.

The immune system is highly regulated by a series of stimulatory and inhibitory pathways that are crucial to maintain a healthy and balanced system. Disruption of the control of this immunological balance can result in abnormal stimulatory signals associated with the loss of immune tolerance in autoimmunity or in the creation of aberrant immunosuppressive networks that occur in cancer. Accumulating evidences have been demonstrating the importance of glycans and glycans binding proteins [including galectins (1, 2), C-type lectins (3), and sialic acid-binding immunoglobulin-type lectins (siglecs) (4, 5)] in the regulation of both innate and adaptive immune responses. In fact, all cells are covered with a dense coat of glycans that constitute a major molecular interface between cells and their environment. The diversity of glycans presentation at cell surface is enormous, encoding a myriad of important biological information that remains to be fully characterized. Glycosylation is the enzymatic process responsible for the attachment of glycans (carbohydrates) to proteins or lipids (predominantly via nitrogen (*N*) and oxygen (*O*) linkages), a process that occurs in the Endoplasmic Reticulum/Golgi compartment of essentially all cells being mediated by the coordinated action of a portfolio of different glycosyltransferases and glycosidases enzymes (6). The proper development and function of the immune system relies both on the dynamic regulation of the expression of glycan-structures and glycan-binding proteins, and the interactions between them (7). This review discusses the role of glycans (with a focus on *N*-linked glycans) on T cells biology and function, including T cell development, activation, differentiation, and signaling. This dynamic interplay between glycans and T cells activity controlling both auto-reactivity and self-tolerance will be presented and discussed (Figure 1).

## Glycans in T cell development and thymus selection

**Figure 2:**
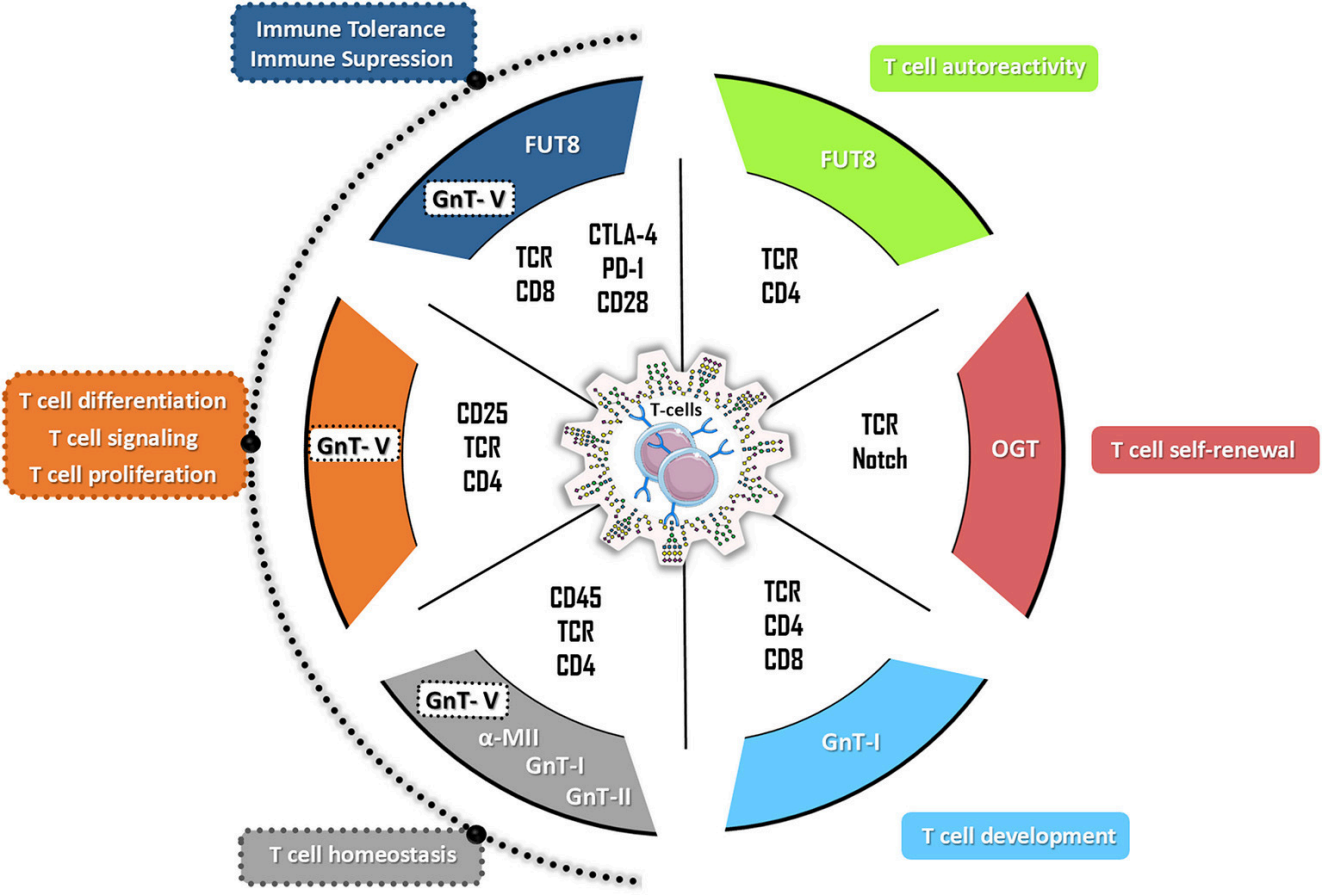
The hallmarks of glycans in T cell biology. *N*-glycans have a broad effect on the multiple T cell functions with impact both in autoreactivity and in immune tolerance. Particularly, the complex branched *N*-glycans catalyzed by beta 1,6-*N*-acetylglucosaminyltransferase V (GnT-V) (encoded by *MGAT5* gene) have been demonstrated to control different T cells functions by targeting different T cells receptors (such as TCR, CD25, and CD4) and therefore regulating T cell proliferation, T cell differentiation, T cell signaling as well as the production of inflammatory cytokines. Alterations on GnT-V activity but also in alpha-mannosidase II (α-MII) as well as in *N*-acetylglucosaminyltransferase I (GnT-I, *MGAT1* gene) and II (GnT-II, *MGAT2* gene) activity were shown to compromise T cell homeostasis being associated with the development of several autoimmune disorders in humans and mouse models (such as EAE, IBD, SLE, TID). The FUT8-mediated core fucosylation of TCR was associated with hyperactivation of CD4^+^ T cells (T cells autoreactivity) whereas the modification of the co-inhibitory receptors (CTLA-4 and PD-1) by FUT8-mediated core fucose results in immune tolerance. The T cell development and T cell self-renewal are controlled by GnT-I-mediated glycosylation and by *O*-GlcNAcylation through OGT (*O*-GlcNAc transferase), respectively.

T cells are developed in the thymus where a microenvironment is set, which enables the selection of T cell receptors (TCRs) to generate a diverse repertoire of potential antigen recognition (8). Lymphoid progenitors from the bone marrow enter into the cortical tissue of the thymus, where they start to expand and develop (9, 10). Despite the fact that the role of glycosylation in T cell development and thymus selections still remains to be fully understood, some important findings highlight the relevance of glycans in this process (Figure 2).

### Role of glycans in thymus seeding and T cell lineage commitment

The initial step of T cell development, the trafficking of thymus-seeding progenitors (TSPs) to the thymus, is an active process that relies on the expression of P-selectin in the thymic epithelium and its partner, P-selectin glycoprotein ligand-1 (PSGL-1), expressed by circulating TSPs-derived from the bone marrow (11). The expression and post-translational modifications of PSGL-1 are regulated in bone marrow progenitors. The deficiency of α1,3 fucosylation on PSGL-1, required for its binding to P-selectin, was shown to be associated with the impairment of TSPs homing into the thymus (12). Once TSPs enter the thymus, they develop into early thymocyte progenitors (ETPs), a subset of the CD4^−^CD8^−^ double negative 1 (DN1) population, which give rise to multiple lymphoid lineages (8). The conserved Notch signaling pathway is responsible for the commitment of DN1 thymocytes to the T cell lineage (13). The glycosylation profile of Notch receptors (and ligands) was shown to regulate Notch-dependent intracellular signal transduction. The lunatic, manic, and radical Fringe are the glycosyltransferases that modify Notch receptors by transferring *N-*acetylglucosamine (GlcNAc) to *O*-linked fucose glycans of epidermal growth factor-like (EGF-like) repeats, present in the extracellular domain of Notch, and described to regulate its cell-surface signaling and function (14, 15). Loss of the three Fringe glycosyltransferases leads to a reduced binding of Notch to Delta-like ligands (DLL), namely DLL4, altering the frequencies of several T cell subsets in the thymus (16). The first indication that Fringe-mediated Notch glycosylation was involved in T cell development was shown when the lunatic Fringe gene, *Lfng*, was misexpressed under a *lck*-proximal promoter (17). This alteration of the Notch glycosylation profile (lack of GlcNAc in the EGF-like repeats) resulted in a large B cell population developed from lymphoid progenitors in the thymus. In fact, further work showed that *Lfng* is poorly expressed in CD4^+^CD8^+^ double positive (DP) thymocytes, but when ectopically expressed in that population (under *lck*-proximal promoter), led to an increased binding of Notch to its ligands on stromal cells, blocking DN development, and enabling B cell differentiation (18). These studies also revealed that changes in the glycosylation of Notch across T cell development also impacts on its signaling pathway. At DN stages, the reactions that drive development are dependent on Notch interactions with DLLs, which exist at functionally limiting concentrations. The high levels of *Lfng* expression in DNs facilitate Notch interactions with DLLs and the dramatic downregulation of *Lfng* in DPs coincides with Notch-independent reactions of T cell development. The final commitment to the T cell lineage occurs at the DN3 stage, where a recombination-activating genes (RAG)-mediated productive rearrangement of the *Tcrb* leads to the expression of the ß chain of the TCR (TCRß) and the formation of a pre-TCR signaling complex (13, 19).

### Role of glycans in thymocyte ß selection

Together with Notch and Interleukin (IL)-7, the pre-TCR signaling initiates ß-selection, by inducing the downregulation of the RAG complex expression (*Rag1* and *Rag2*) in quiescent DN3 (DN3a), becoming large cycling DN3 thymocytes (DN3b), which differentiate into DN4 cells. A deficient pre-TCR signaling in *lck*-null cells is rescued by *Lfng* overexpression, but not in a *Rag2*^−/−^ background, indicating a pre-TCR dependency for development (20). Upon ß-selection, it was recently demonstrated that DN4 cells upregulate glucose and glutamine metabolites that enter into the hexosamine pathway, increasing the production of UDP-GlcNAc, which is needed to undergo clonal expansion (8, 21). The UDP-GlcNAc is also the substrate of the *O*-GlcNAc transferase (OGT) in the process of *O*-GlcNAcylation of intracellular proteins on serine and threonine residues (22). Recent evidences showed that *O*-GlcNAcylation regulates the process of T cell development (23). Using a conditional knockout mouse model of OGT in the DN stage, it was shown a reduced population of DPs, indicating either a deficiency on ß-selection or in clonal expansion of DN4s. The absence of OGT appeared not to impact self-renewal of DNs, or their differentiation into DPs, but to promote the failure of the clonal expansion of DN4, in response to Notch ligands. A feedback mechanism was proposed in which the metabolic changes (the shift to glycolysis) that support the DN-to-DP stage of thymocyte differentiation, controlled by Notch, induces c-Myc expression, which in turn controls the rate of T cell nutrient uptake as well as the expression of OGT and consequently the abundance of *O*-GlcNAc (15). The *O*-GlcNAcylation of c-Myc was also shown to increase its stability (24), further contributing to the feedback loop.

In the stage of post-β selected DN4 thymocytes, it was seen a 10-fold increase in expression of ST6 β-Galactoside α2,6-Sialyltransferase 1 (ST6Gal I) when comparing to the DN3 population, which resulted in an increase in α2,6-linked sialic acid (25). Accordingly, in *ST6Gal1* deficient mice, the DN populations were decreased, beginning at the DN1 subset. Microarray data showed a downregulation of CD96 (receptor molecule of nectin-1, that plays a putative role in cell migration) in the DN2 and DN3 populations in the *ST6Gal1* deficiency background, and a disruption of thymopoiesis in these mice was proposed. Moreover, ST3 β-Galactoside α2,3-Sialyltransferase 1 (ST3Gal I) expression is decreased in most DN and in all DP, only increasing in single-positive (SP) thymocytes (26). In *ST3Gal1*^−/−^ mice, the TCR repertoire was significantly altered, indicating a role for sialylation in thymocyte selection (27).

### Role of glycans in positive and negative selection in the thymus

The ß-selected DN4 cells undergo rapid self-renewal, giving rise to a clonally expanded population, that differentiate into DP CD4^+^CD8^+^ thymocytes (8). In this developmental stage, mature TCRαß receptors are formed (28) and the expression of the co-receptors CD4 and CD8 confer a MHC class II and class I restriction of TCR activation, respectively. The newly formed mature TCRs are then screened by thymic epithelial cells (TECs) by the specificity and binding strength for the MHC ligands presented. The next developmental process is named positive selection, where the DP population is enriched for cells that express an immunocompetent TCR (8, 29). The selected DPs then commit to the SP CD4^+^ or CD8^+^ lineage and go through a process called negative selection, which eliminates autoreactive T cells (8, 29). The affinity of the correctly assembled TCRαβ for the MHC-antigen complexes determines cell survival and differentiation. Glycosylation modifications of the TCR may provide an alternative mechanism to control positive and negative selection by directly affecting the TCR-MHC-antigen binding, TCR interaction with its co-receptors and the threshold of activation (30), an issue that is far from being fully elucidated. The subunits of the TCRαβ contain at least 7 potential sites for *N-*linked glycosylation and the TCR-CD3 complex is estimated to have 12 *N*-glycan addition sites that contribute to TCR folding and functions (31, 32). Indeed, selective removal of conserved *N-*glycosylation sites of the constant regions of the TCR, enhanced its functional avidity (the sensitivity of the T cell response to other cell which carries the respective MHC-peptide) (32). However, whether *N-*glycosylation in the variable regions of the TCR affect its selection remains to be addressed. Moreover, low levels of sialylation in DPs are associated with binding to Major Histocompatibility Complex (MHC) class I (common to all nucleated cells) and the increased expression of sialic-acid linkages on differentiated SP CD8^+^ thymic T cells was shown to decrease the binding avidity of CD8 for MHC class I molecules, which acts as a regulation for a TCR affinity dependent negative selection (33).

Furthermore the deficiency of the *Mgat5* gene, that encodes for a Golgi branching enzyme *N-*acetylglucosaminyltransferase V (GnT-V) was shown to markedly increases TCR clustering and signaling at the immune synapse, resulting in a lower T cell activation threshold and increased incidence of autoimmune disease *in vivo* and in human (30). In a model of positive selection, it was demonstrated that branching *N-*glycosylation dynamically expands the affinity spectrum of positive selection by differentially controlling both the lower and upper limits of positively selected TCR-MHC-antigen interactions (34). The intracellular domains of CD4 and CD8 co-receptors bind Lck, enhancing TCR responses to low-affinity MHC-antigen complexes when coupled to the TCR (35). Both co-receptors have *N-*glycosylation sites and it was shown that the branching deficiency in Mgat1^f/f^Lck-Cre^+^ T cells resulted in decreased surface expression of CD4 and CD8 receptors (34). The lack of branched *N-*glycans in the same genetic background also decreased TCR threshold signaling (30). These evidences supported that branching *N-*glycans display an important role in the maturation of DN cells and/or TCR selection.

Changes in the expression of *O*-linked glycans also impact T cell development by modulating galectin binding. Galectin-1 was shown to induce apoptosis of immature thymocytes through binding to core 2 *O*-glycans expressed in CD43 and CD45 (36). In contrast, CD45 on mature thymocytes bears core 1 *O*-glycans as well as *N-*glycans capped with α2,6-linked sialic acid, which inhibits galectin-1 binding (36).

Overall, glycosylation appears to play a critical role in the different stages of thymocyte development and in the generation of an efficient immune system. Nevertheless, further research is needed in order to understand how glycans control each stage of thymocytes development, differentiation and selection, which might reveal novels insights on the influence of the glycome in major diseases, such as autoimmunity and cancer.

## Glycans in the regulation of T cell activity and functions

The proper function of T lymphocytes is highly dependent on their surface receptors, which in turn are highly mediated by glycosylation. Although *O*-glycan structures have been shown to play important roles on immune-associated molecules (37), the prominent role of *N-*linked glycans is emphasized in this section (Figure 2).

As previously mentioned, MHC I is expressed by almost all nucleated cells and interacts with TCRs on CD8^+^ T cells; in turn, MHC II is expressed by professional antigen presenting cells (APCs) (dendritic cells - DC, macrophages, B cells and TECs) and is recognized by CD4^+^ T cells (7, 38). More than 3 decades ago, it was demonstrated that blocking MHC1a *N-*glycosylation, through acceptor site mutation, results in significant increases in intracellular misfolded protein along with decreases in cell surface expression (39). MHC II is assembled by two glycoproteins, α and β chains. The α chain contains *N-*linked high-mannose and complex glycans whereas the β chain is only constituted by complex *N-*glycans (40). In contrast to the role of MHC I, MHC II glycosylation was shown to have a particular impact on the effective antigen binding, as well as in the presentation of microbial carbohydrate antigens, which consequently influences downstream T cell responses. This was demonstrated by the depletion of the *Mgat2* gene, which compromises *N-*glycan branching, decreasing carbohydrate antigen presentation by MHC class II and leading to loss of T cell stimulatory activity (41).

During TCR signal transduction, glycans play a key role in stabilizing individual molecules in the complexes at the immunological synapse and by protecting them from the action of proteases during T cell engagement (31). Additionally, glycans can also restrict nonspecific protein-protein interactions, like aggregation of TCRs on the membrane, helping to orient the interactions of the proteins in the central clusters (31). Demetriou et al. demonstrated that β1,6-GlcNAc branched *N-*glycans structures (catalyzed by GnT-V) regulate T cell activity, namely in CD4^+^ T cells by increasing the threshold of T cell activation, suppressing T cell growth and signaling (30, 42). Moreover, core-fucosylation, which refers to fucose attached to the innermost *N-*acetylglucosamine of *N-*linked glycans, catalyzed by α1-6 fucosyltransferase (FUT8), was also shown to affect T cell activity in immune mediated disorders (42, 43).

The T cell activity is also dependent on glycosylation of co-receptors, such as the complex formation between TCR and CD45. Galectin-3 is a key mediator of this complex, by establishing a molecular lattice through binding to polylactosamine structures in branched *N-*glycans. Consequently, CD45 phosphatase activity induces downregulation of T cell signaling, preventing T cell activation (44). Furthermore, CD45 is alternatively spliced into five different isoforms on human leukocytes (CD45ABC, CD45AB, CD45BC, CD45B, and CD45RO) (45–47), all decorated with up to 11 *N-*glycans in the membrane proximal region. Importantly, all isoforms present different glycosylation profiles (48, 49), that change during T cell differentiation and activation (50, 51), as reviewed in (36). CD28 is another T cell surface glycoprotein acting as a secondary signaling molecule of T cell activation. Interestingly, nearly 50% of the molecular mass of CD28 is constituted by *N-*glycans (52). Previous studies reported that *N-*glycosylation of human CD28 can negatively regulate CD28-mediated T cell adhesion and co-stimulation, namely the interaction between CD28/CD80. Mutation of all potential *N-*linked glycosylation sites of CD28 as well as treatment of Jurkat cells with inhibitors of *N-*glycosylation pathway resulted in a defective CD28 glycosylation with enhancement of the binding to CD80 expressed on APCs (52). The branching *N-*glycosylation of CD25 receptor also modulates its cell surface retention controlling T differentiation with impact in immune tolerance. Recently, it was demonstrated that a decreased UDP-GlcNAc and complex branching *N-*glycosylation induces a decreased cell surface retention of CD25 and IL-2 signaling, promoting a T helper (T_H_) 17 over induced regulatory T cell (iTreg) differentiation (53) (Figure 2).

Importantly, the co-inhibitory receptors are likewise modulated by *N-*glycosylation. One of the major negative regulators of T cell response is the cytotoxic T-lymphocyte protein 4 (CTLA-4), that comprises two *N*-glycosylation sites described to modulate its cell surface retention on T cells and thereby its affinity for CD80/CD28 on APCs (54–56). The impact of *N-*glycosylation in the modulation of the inhibitory functions of CTLA-4 and programmed cell death protein-1 (PD-1) is discussed in more detail in section “Glycans in tolerogenic/immunosuppressive responses”. Nonetheless, other co-inhibitory receptors like Lymphocyte-activation gene 3 (Lag-3), mucin-domain-containing molecule-3 (Tim-3), and T cell immunoreceptor with Ig and ITIM domains (TIGIT) may also undergo glycans-mediated regulation, as they exhibit *N-*glycan-binding sites, however the role of glycans on these molecules remains to be explored (57).

Taken together, *N-*glycosylation plays an instrumental role in the regulation of T cell activation and functions by targeting not only TCR but also its co- receptors (Figure 2).

## Glycans as modulators of hyper-reactive/autoimmune responses

Autoimmunity is characterized by the loss of self-tolerance and development of an autoreactive immune response toward the individual's own organism. Glycan motifs play a crucial role in the determination of self/non-self antigens. Specific glycan structures, expressed by microbial pathogens, are commonly responsible for the primary activation of the innate immune system; however, the mechanisms involved in the self/non-self discrimination, mediated by glycans are far from being fully elucidated. Abnormal levels of branched *N*-glycans have been associated with exacerbated immune responses in murine models (58). Particularly, the dysregulation of the *N*-glycosylation pathway has been associated with autoimmune-like phenotypes. The inability to synthetize β1,6-GlcNAc antennae, in *Mgat5*^−/−^ mice has been associated with an increased susceptibility to immune-mediated disorders such as an increased delayed-type hypersensitivity responses, as well as increased susceptibility to develop experimental autoimmune encephalomyelitis (EAE) (30, 59) and severe forms of colitis (60). The lack of β1,6 branching *N*-glycans favors TCR clustering, leading to a decrease of the TCR threshold and consequently increased T cell activation (30) associated with the hyperimmune response observed in these mice (Figure 2). This hyperimmune phenotype is also due to an abnormal formation of lattices between TCR-branched glycosylation and galectins (61, 62). Accordingly, β3 GnT2-deficient mice show T cell hypersensitivity due to the reduction of polylactosamine on the *N*-glycans (ligands of galectins), similarly to what is observed in *Mgat5* deficient mice (30, 61). Furthermore, absence of α-mannosidase II (which catalyses the last hydrolysis of the α-mannose), was shown to result in signs of glomerulonephritis, deposits of glomerular IgM immunocomplexes and complement component 3 as well as high levels of anti-nuclear antibodies (63, 64), which is consistent with a Lupus-like syndrome (Figure 2). Taken together, these evidences support the role of *N*-glycosylation in the perspective of T cell biology.

The role of *N*-glycans in antigen presentation and recognition is still elusive, and in fact abnormal glycoantigen presentation might also impact T cell activity. Abnormal accumulation of high-mannose, paucimannose, and agalactosyl bi-antennary glycans, have been detected in kidney tissue from MRL-lpr mouse (a well-stablished murine model of SLE) (65). Moreover, evidences have been showing that *Mgat1*^f/f^Syn1-Cre mice, with *Mgat1* deletion at the Synapsin I*-expressing cells* (abundant in neural tissues), presented neurological defects, with high levels of neuronal apoptosis and caspase 3 activation (66). These high levels of apoptosis are observed in several autoimmune diseases, which results in activation of immune system (67) (Figure 2). Although highly unexplored, rare autoimmune diseases are also associated with *N*-glycosylation dysfunctions. As example, idiopathic inflammatory myopathies (IIM) are a group of rare diseases of autoimmune nature, whose etiopathogenesis is far from being totally understood (68). Muscle cells surface is enriched with glycoproteins and several lines of evidence provide support for a fundamental role of glycosylation in muscle homeostasis and function (69, 70). Glucosamine (UDP-*N*-Acetyl)-2-Epimerase/*N*-Acetylmannosamine Kinase (GNE) genetic mutations (a gene that encodes *N*-acetylmannosamine (ManNAc) kinase enzyme, responsible for the biosynthesis of *N*-acetylneuraminic acid) results in hypo-sialylation of muscle glycoproteins; the prophylactic supplementation with sialic acid precursor (ManNAc) was shown to prevent the muscle phenotype in mice with gene mutations that cause hereditary inclusion-body myositis (hIBM), a muscle phenotype that resembles one type of IIM (71). Altogether, these findings highlight the importance of further studies addressing the role of *N*-glycosylation in the perspective of *neoautoantigens*, since autoantigens contain a significant amount of glycoantigens due to the increased number of *N*-glycosylation sites comparing with other proteins (72).

The Glycan binding proteins (GBPs) are expressed in the APCs being characterized by a carbohydrate recognition domain which specifically recognizes glycan structures present at the cell surface receptors. This glycan-GBPs engagement results in either an anti- or pro-inflammatory response (73). C-type lectins, siglecs, and galectins are examples of GBPs, that are instructors of immune responses (5, 73). As example, SIGN1R (expressed by APCs and the analogous of the human dendritic cell-specific ICAM-grabbing non-integrin - DC-SIGN) signaling was shown to result in the expansion of IL-10-secreting Treg cells, preventing the development of autoimmune diseases such as EAE and type 1 diabetes (T1D) (74). Galectin-1 also plays an important immune-regulatory role in EAE (75) as mice deficient in galectin-1 (*Lgals1*^−/−^) have increased T_H_1 and T_H_17 responses being more susceptible to EAE when compared with wild type mice (76). More recently, Galectin-1 was shown to modulate the cytolytic activity of CD8^+^ T cell. The interaction of Galectin-1 and Fas ligand seems to be responsible for the retention of this glycoprotein at the surface of cytotoxic T lymphocytes hampering the cytolytic ability of these cells (77). Overall, GBPs-glycoprotein interaction is essential to instruct a T cell—mediated immune response.

Notably, one of the first evidences addressing the relationship between the dysregulation of *N*-glycosylation and human autoimmunity was observed in multiple sclerosis (MS) patients. During active, relapse or in very early stages of remission, peripheral blood mononuclear cells from MS patients display a significant decrease of the enzymatic activity of Golgi β1,6 *N*-acetylglucosaminyltransferase (core 2 GlcNAc-T), compared to healthy subjects (78). Moreover, *MGAT5* polymorphisms were associated with MS severity (79) together with *MGAT1, IL2R*, and *IL7R* Single Nucleotide Polymorphisms (80–82). Additionally, in Inflammatory Bowel Disease (IBD), it was also demonstrated that *lamina propria* T lymphocytes from ulcerative colitis (UC) patients exhibited a deficiency in β1,6-GlcNAc branching *N*-glycans due to decreased levels of *MGAT5* gene expression (83). Importantly, low levels of branched *N-*glycans in lamina propria early at diagnosis were shown to predict UC patients that will fail the response to standard therapy, thus displaying a bad disease course (84). The supplementation of intestinal T cells from UC patients and mouse models with colitis with GlcNAc promoted the enhancement of β1,6 branching *N*-glycans on T cells, suppressing TCR signaling and reducing the production of pro-inflammatory cytokines such as tumor necrosis factor alpha (TNFα) and interferon gamma (IFNγ). Pre-clinical studies both in IBD and MS demonstrated the immunomodulatory properties of *N*-glycans in the control of T cell-mediated immune response (60, 85), paving the way for the development of human clinical trials, that are currently on going (53, 60). Less explored but of utmost importance is the study of *N*-glycosylation profile in rare autoimmune disorders, since its etiopathogenesis is still very elusive. Glycosylation changes in muscle-associated human disease have focused in muscular dystrophies (86) and congenital disorders of glycosylation (87). Recent studies have shown that muscle cell surface glycosylation is finely regulated and subjected to alterations under inflammatory conditions (88), pointing to a possible interaction between muscle glycocalyx and the extracellular milieu, which is particularly enriched in immune cells and antibodies in IIM patients (89).

Overall, glycans are critical determinants in autoreactive responses both by directly regulating T cell activity and also through the creation of abnormal glycoantigens that may unleash an autoreactive immune response.

## Glycans in tolerogenic/immunosuppressive responses

Recent studies have been highlighted that alterations on the glycosylation pattern of T cells' receptors, as well as the alterations of the glycosylation profile of tumor cells (tumor glyco-code), are implicated in the modulation of the immune response leading to immunosuppressive pathways, known to occur in the tumor microenvironment associated with tumor immunoescape (90).

### Role of glycans in the modulation of inhibitory T cell receptors

PD-1, as already introduced, is a cell surface inhibitory T cell receptor responsible for immune-inhibitory responses associated with the so-called “T cell exhaustion” (91). The expression of this cell surface receptor, as well as Tim-3, was described to be positively regulated by the core fucosylation pathway, catalyzed by FUT8 enzyme (92). The inhibition of core fucosylation in PD-1 was demonstrated to lead to an anti-tumor immune response mediated by T cells activation, being a new attractive target for enhancing anti-tumor immunity in future clinical settings (Figure 2). This was a pioneer study that supported the importance of PD-1 post-translational modifications by glycosylation on T cell-mediated immunosuppression (92). Additionally, the glycosylation of programmed death ligand-1 (PD-L1), a PD-1 ligand, was described to have an important role in its cellular stabilization. The interaction of non-glycosylated PD-L1 with glycogen synthase kinase 3β (GSK3β), a key enzyme on glycogenesis, leads to the degradation of this molecule (93). In triple-negative breast cancer cells, it was further shown that the β1,3-*N*-acetylglucosaminyl transferase (B3GNT3), involved in the biosynthesis of poly-*N*-acetyllactosamine chains, is important for the interaction between PD-1 and its ligand PD-L1 (94). The use of an antibody targeting the glycosylated form of PD-L1 resulted in its degradation and internalization, with the blockage of PD-L1/PD-1 interaction and consequently the inducement of anti-tumor activity in triple-negative breast cancer *in vitro* and *in vivo* models (94). In accordance, Tregs from healthy humans and mice were shown to display an increased variability on its *N*-glycosylation pattern when compared with CD4^+^ T cells. The levels of the complex branched *N*-glycans were shown to be correlated with the expression of proteins involved in Treg suppressive functions, including PD-1, PD-L1, and also other negative regulators of T cell response, namely CTLA-4 (95). In fact, the CTLA-4 protein, comprises multiple *N*- and *O-*glycosylation sites known to modulate its retention at T cell surface and consequently affecting its function (56). The TCR activation is associated with an increased β1,6-GlcNAc branched *N*-glycosylation of CTLA-4, which enhances CTLA-4 retention at the T cell surface and thereby suppresses T cell activation promoting immune tolerance (96) (Figure 2). Accordingly, the presence of Thr17Ala polymorphism in human *CTLA-4* was shown to result in the reduction of the *N*-glycosylation sites from one to two sites, which limited CTLA-4 retention at T cell surface (80). Supplementations with GlcNAc and Vitamin D promoted an enhancement of *N*-glycans branching expression, increasing the cell surface retention of CTLA-4, culminating in immunosuppression (80).

### Glycans as instructors of immunosuppressive responses

Tumor cells aberrantly express different types of glycans structures when compared with normal counterparts, such as an increased sialylation, an expression of truncated glycans and an overexpression of branched *N*-glycans (97). This alteration in the cellular glycosylation profile governs several steps of tumor development and progression, such as tumor cell dissociation, proliferation, invasion, metastasis, angiogenesis, with recent evidences pointing toward its effects in tumor immunoediting and immunosurveillance (98). GBPs expressed on immune cells are able to recognize altered glycan structures expressed at tumor cell surfaces instructing either immunostimulatory or immunoinhibitory responses.

The expression of sialylated glycans, such as Tn antigen and Lewis antigens, aberrantly expressed in tumor cells, were described to be recognized by DC-SIGN, expressed by macrophages and immature DCs, which lead to immunosuppression (99). The fucose residues present in Lewis structures (Lewis x and Lewis y), attached to carcinoembryonic antigen (CEA) (100), were described to trigger the upregulation of the anti-inflammatory cytokines IL-10 and IL-27 by APCs and the induction of T_H_2, follicular (T_H_f), and Treg immune responses (101, 102). Besides, antigen-containing liposomes modified with DC-SIGN-binding Lewis b and x resulted in glycans recognition and internalization through DCs with consequent activation of CD4^+^ and CD8^+^ T cells (103). Furthermore, macrophage galactose binding lectin (MGL) was found to be able to recognize Tn antigen and *N*-acetylgalactosamine (GalNAc) residues, resulting in an increased recognition by Toll-like receptor 2, ultimately resulting in the secretion of cytokines (IL-10 and TNF-α). (104). Its interaction with terminal GalNAc residues on CD45 glycoprotein negatively regulates TCR signaling, with consequent decrease of T cell proliferation and increased T cell death (105). Moreover, by blocking the tumor-infiltrated macrophages (responsible for the high levels of IL-10), it was observed an effective CD8^+^ T cells response, highlighting the importance of combining anti-tumor immune therapy with conventional chemotherapy (106). Furthermore, it was recently demonstrated in chronic infection that IL-10 induces the upregulation of the *Mgat5* gene increasing branched *N*-glycans on CD8^+^ T cells, which in turn decreases T cell activity and allows viral persistence (107). Despite the different context in which this hypothesis was studied, *Mgat5-*mediated branching glycosylation can constitute a potential mechanism by which IL-10 is suppressing CD8^+^T cells in cancer.

In addition, sialylated glycans also play a role in immunosuppression, mediated by siglecs, a family of lectin receptors that predominantly exhibit immune-inhibitory functions. In *in vitro* and *in vivo* studies, the binding to sialylated antigens by siglec-E expressed on DCs promoted an increase of antigen-specific Treg response and a reduced numbers of antigen-specific Teff cell response, associated with tumor growth (108, 109). Indeed, the sialylated tumor antigens, such as Sialyl-Tn (sTn) and Sialyl-T (sT) expressed in mucins, namely MUC1, were associated with tumor immune tolerance. The recognition of MUC1-ST by siglec-9 on tumor-infiltrating macrophages was shown to initiate inhibitory immune pathways mediated by MEK-ERK signaling (110). Moreover, siglec-binding to sTn-expressing mucins, led to the maturation of DCs and DC-mediated induction of FOXP3^+^ Treg cells and reduced INFγ-producing T cells (111, 112). A recent study also demonstrates that siglec-9 expressed by CD8^+^ tumor infiltrating lymphocytes (TILs) in non-small cell lung cancer (NSCLC) patients was associated with reduced survival. Accordingly, siglec-9 polymorphisms were associated with the risk of developing lung and colorectal cancer. Additionally, the characterization of siglec-9^+^ CD8^+^ TILs revealed that these cells concomitantly express several inhibitory receptors, including PD-1, TIM-3, Lag3, and others. In addition, the same study further reveals that lack of sialic acid-containing glycans in tumor cells led to a delay of tumor growth and an increased infiltration of CD3^+^ and CD8^+^ T cells (113).

Another important GBP that have been pointed out as a crucial checkpoint in T cell viability and activity are galectins. Galectin-1, 3, and 9 were predominantly described in T cell immunosuppression. Galectin-1, was demonstrated to be expressed by tolerogenic DCs (75) and CD4^+^CD25^+^ T cells (114), triggering T cell apoptosis through binding to *N*-glycans and *O*-glycans on CD45, CD43, and CD7 or by sensitizing resting T cells to FAS-induced death (115, 116). The T_H_1 and T_H_17 activated cells are susceptible to galectin-1-induced cell death once these cells express the repertoire of glycans required for galectin-1 binding, while T_H_2 cells are protected via α2,6-sialylation on cell surface glycoproteins, which was described to preclude galectin-1 recognition and binding (76). In addition, several tumors have the capacity to secrete galectin-1 in order to promote immunosuppression, through a mechanism that involves a bias toward a T_H_2 cytokine profile and activation of tolerogenic circuits mediated by IL-27-producing DCs and IL-10-producing type 1 Treg cells (117). On other hand, galectin-3 has an ambiguous role in T cell viability: when it is localized at intracellular level, this protein presents a protective role through a cell death inhibition pathway that involves B-cell lymphoma 2 (Bcl-2) (118), whereas extracellular galectin-3 induces cell death in activated T cells, by binding to glycosylated receptors of T cells through a distinct way than galectin-1 (115). Moreover, galectin-3 has the capacity to bind to *N*-glycans on CTLA-4 prolonging the inhibitory signals (119), as well as to Lag-3 on the surface of CD8^+^ T cells, suppressing its function (120). Finally, galectin-9 abrogates T_H_1, T_H_17, and CD8^+^ T cells through glycosylation-dependent binding to Tim-3 (121–123), whereas may regulate pro-inflammatory cytokine production by binding with other receptors (124).

Altogether, these findings support the relevance of glycans on T cells-mediated immunosuppressive/tolerogenic pathways which have relevant implications in tumor progression. Targeting the abnormal glycosylation pattern of cancer cells constitutes a promising strategy to instruct an effective anti-tumor immune response, an issue that needs to be further explored.

## Glycans as metabolic regulators of T cell function

The impact of glycosylation on T cell development and functions is enormous, as revealed by the critical roles of glycans in the development and progression of major diseases such as auto-immunity and cancer, as described herein. In order to accompany the bioenergetic and biosynthetic demands required for T cell proliferation and activation, a shift in the T cell metabolism is required. While naïve T cells are in a metabolic quiescent state, mainly using oxidative phosphorylation to maximize ATP production, T cells under clonal expansion or under differentiation, reprogram their metabolic status to aerobic glycolysis and glutaminolysis in order to increase the availability of glycolytic precursors for the biosynthesis of nucleotides, amino acids and lipids (125–127). During T cell activation, the hexosamine biosynthetic pathway (HBP—a branch of the glucose metabolism) is upregulated in order to generate the nucleotide sugar-donor substrate UDP-GlcNAc, required for *N-*glycosylation, O-GlcNAcylation, and glycosaminoglycans production that are needed for a proper T cell function (128).

Mediators from the glycolytic pathway such as glucose (Glc), glutamine (Gln), acetyl CoA are known to interfere with the availability of the UDP-GlcNAc in the cell (129–131). Together Glc and Gln were shown to increase UDP-GlcNAc in nutrient-starved T cells. In the same setup, the supplementation of both Glc and glucosamine (GlcN–a metabolite of the HBP) further increased the UDP-GlcNAc cellular content, demonstrating the sensitivity of the HBP to nutrients that enter directly (GlcN) or through a precursor pathway (Glc in glycolysis) (130). Despite the general use of the UDP-GlcNAc as a substrate donor of HBP, there are some glycosyltransferases that are more susceptible to nutrient changing than others, such as the case of OGT (132). In fact, the supply with Glc and Gln are crucial for protein O-GlcNAcylation, that is important during T cell development, being associated with T cell malignant transformation (23). Among the *N-*acetylglucosaminyltransferases (GnTs) that participate in the HBP, the less sensitive to nutrient changing (and thus substrate availability) are GnT1, GnT2, and GnT3, due to lower *Michaelis Constant (K*_*m*_*)* levels, meaning that these enzymes require low levels of the substrate to synthetize the specific glycans. In contrast, GnT4 and GnT5 present higher *K*_*m*_ and therefore their activity is highly dependent on the availability of the UDP-GlcNAc substrate (119, 133). Therefore, these two enzymes are sensitive to alterations in glucose and HBP metabolism (as the GlcN or *N*-acetyl glucosamine–GlcNAc) (62), which ultimately will interfere in the *N*-glycan branching biosynthesis on T cells with impact in their activity, as detailed in section “Glycans in the regulation of T cell activity and functions” (60). In fact, supplementation with Glc, Gln, and GlcNAc increases branching *N*-glycans on Jurkat cells and resting T cells from mice (85, 119, 130). Moreover, CD4^+^ T cells from MGAT5^+/+^ or MGAT5^+/−−^ mice supplemented with oral GlcNAc also results in up to 40% increase of branching *N-*glycans, detected by L-PHA (130). This enhancement of branching *N-*glycosylation upon GlcNAc supplementation was shown to functionally impact on T cells activity by reducing T cell activation, decreasing T_H_1 differentiation, and increasing retention of the growth inhibitory receptor CTLA-4 at T cell surface (85, 130).

Importantly, evidences suggest that the glycolysis and glutaminolysis compete with HBP pathway for the same metabolites. Recently, Araujo et al showed that, during T_H_17 differentiation the existence of common mediators shared between HBP, glycolysis (fructose-6-phosphate) and glutaminolysis (Gln) results in a starvation of the HBP mediators, translated in a reduction of *N-*glycan branching due to the limitation on the UDP-GlcNAc availability (53). Fueling HBP with GlcNAc switched the cell fate from T_H_17 to iTreg differentiation, through stimulation of IL2-Rα signaling (53). This interplay between metabolic pathways was further demonstrated by the increase on Glc, Gln, fatty-acids uptake, and lipid storage upon stimulation of the HBP with GlcNAc supplementation, suggesting a reprogramming of the cellular metabolism upon GlcNAc flux (53, 134).

The impact of glycans as metabolic regulators of T cells is also testified by its effects in *ex vivo* and *in vivo* models of autoimmune diseases. The metabolic supplementation with GlcNAc in *ex vivo* human colonic T cells from IBD patients resulted in an enhancement of the branching *N*-glycosylation pathway that was accompanied by a significant reduction of T cell proliferation, supression of T_H_1/T_H_17 immune response (through decreased production of IFN-γ and IL-17A pro-inflammatory cytokines) and decreased TCR signaling (60). Accordingly, the GlcNAc supplementation of mice models with auto-immune diseases such as EAE, TID, and IBD results in inhibition of T_H_1, T_H_17 immune response concomitantly with a significant improvement of the clinical symptoms (60, 85). Treatment with GlcNAc after disease onset also demonstrate inhibitory effects on the development of the EAE, by reducing the secretion of INF-γ, TNF-α, IL-17, and IL-22 (85). Interestingly, a dual role of GlcN (the precursor of GlcNAc) on the progression of autoimmune disorders was shown, by demonstrating its impact in preventing T_H_1-mediated Type I diabetes (through the reduction of IFN-γ producing CD4^+^ T cells), but also the GlcN effects in exacerbating T_H_1/T_H_17–mediated EAE symptoms (trough stimulation of T_H_17 response) (135). In contrast, another study showed that GlcN suppresses acute EAE through the blockage of T_H_1 and induction of T_H_2 response (136). GlcN supplementation was further shown to mediate T cell activation by decreasing the *N-*glycosylation of CD25 (IL-2Rα) from CD4^+^ T cell (135). This down-regulation of *N-*glycosylation might be explained by the competition between GlcN and Glc for the same glucose transporter which might impact in the reduction of the GlcNAc concentration.

Altogether, alterations on the glucose metabolism and partially changes in the metabolic flux of HBP have a direct impact on T cells *N*-glycosylation profile with major consequences in their function and activity. Ultimately, the modulation of the HBP constitutes an important metabolic target able to control both autoreactive and immunosuppressive responses known to occur, respectively, in autoimmunity and cancer.

## Concluding remarks

The contribution of the glycome as a major regulator of the immune system is clear. Glycans actively participate in the cellular and molecular mechanisms underlying the genesis of the loss of immunological tolerance associated with (auto)immunity, from one hand, participating also in the creation of tolerogenic pathways associated with cancer progression, from the other. The importance of glycans in immune response spans from its role in the modulation the T cell development; their importance as a source of glycoantigens presentation; as well as their role as fine tuners of T cell response. In this context, glycans can exert a dual role, acting either as immune inhibitory checkpoints or as immune stimulatory signals. Understanding in depth the influence of glycans in the immune regulatory circuits that mediate the pathophysiology of autoimmunity and cancer will generate a platform with extraordinary potential to illuminate the identification of novel biomarkers and targets for the development of efficient immunomodulatory strategies with applications in the clinical setting.

## Author contributions

All the authors wrote the manuscript. AD and NP created the figures. SP performed the critical review of the manuscript.

### Conflict of interest statement

The authors declare that the research was conducted in the absence of any commercial or financial relationships that could be construed as a potential conflict of interest.
